# The Human Bone Marrow May Offer an IL‐15‐Dependent Survival Niche for EOMES^+^ Tr1‐Like Cells

**DOI:** 10.1002/eji.202451644

**Published:** 2025-05-16

**Authors:** Nadia Pulvirenti, Chiara Vasco, Camilla Righetti, Petra Dadova, Giacomo Boffa, Alice Laroni, Tiziana Vigo, Anna Maria Raiola, Maria Cristina Crosti, Stefano Maglie, Luca Valenti, Daniele Prati, Sergio Abrigani, Antonio Uccelli, Jens Geginat

**Affiliations:** ^1^ Fondazione Istituto Nazionale di Genetica Molecolare Romeo ed Enrica Invernizzi Milan Italy; ^2^ Department of Neuroscience, Rehabilitation, Ophthalmology, Genetics, Maternal and Child Health University of Genova Genova Italy; ^3^ IRCCS Ospedale Policlinico San Martino Genova Italy; ^4^ Ematologia e Terapie Cellulari IRCCS Ospedale Policlinico San Martino Genova Italy; ^5^ Department of Transfusion Medicine Fondazione IRCCS Ca’ Granda Ospedale, Maggiore Policlinico Milan Italy; ^6^ Biological Resource Center Unit Fondazione IRCCS Ca’ Granda Ospedale Maggiore, Policlinico Milan Italy; ^7^ Department of Pathophysiology and Transplantation Università degli Studi Milan Italy; ^8^ Department of Clinical Sciences and Community, Department of Excellence 2023–2027 Università degli Studi Milan Italy

**Keywords:** bone marrow, CD69, memory T lymphocytes, regulatory T‐cells

## Abstract

Maintenance of memory T‐cells in the bone marrow and systemically depends on the homeostatic cytokines IL‐7 and IL‐15. An immunological memory may also exist for regulatory T‐cells. EOMES^+^type‐1 regulatory (Tr1)‐like cells have a rapid in vivo turnover, but whether they are short‐lived effector cells or are maintained long‐term has not been investigated.

EOMES^+^Tr1‐like cells expressing GzmK were enriched among CD69^+^Ki67^−^T‐cells in the bone marrow of healthy donors, suggesting that they became quiescent and bone marrow‐resident. Conversely, CD4^+^GzmB^+^ effector T‐cells were excluded from the bone marrow‐resident fraction. The dichotomy between GzmK^+^ and GzmB^+^T‐cells was observed both in healthy individuals and in multiple sclerosis patients, and also among CD8^+^T‐cells. Intriguingly, bone marrow‐resident CD4^+^ memory T‐cells expressed increased levels of IL‐7Rα, while EOMES^+^Tr1‐like cells were consistently IL‐7Rα^lo^. However, EOMES^+^Tr1‐like cells expressed the IL‐2/15Rβ chain, and the latter was induced upon forced expression of EOMES in primary human CD4^+^ T‐cells. Finally, IL‐15 rescued EOMES^+^Tr1‐enriched populations from death by neglect but was not required for CD4^+^ memory T‐cell survival. These findings suggest that the bone marrow may provide a survival niche for EOMES^+^Tr1‐like cells. The different IL‐7 and IL‐15 receptor expression patterns of CD4^+^ memory T‐cells and EOMES^+^Tr1‐like cells suggest furthermore that they compete for different homeostatic niches.

## Graphical abstract

Human CD8^+^ and CD4^+^ memory and regulatory T‐cell subsets, but not naïve or effector cells, may become CD69^+^ bone marrow‐resident cells, suggesting that they could be maintained long‐term as quiescent cells. CD4^+^ memory T‐cells and EOMES^+^Tr1‐like cells, respectively, expressed IL‐7Rα and IL‐15Rβ, suggesting that they compete for different homeostatic niches.

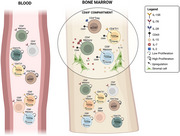

## 1 Introduction

Immunological memory is maintained by memory lymphocytes and plasma cells that can persist in the absence of antigen for a lifetime [1]. Plasma cells are maintained in a quiescent state in specialized niches of the bone marrow [2]. Seminal studies have shown that antigen‐independent survival and proliferation of memory T‐cells are controlled by the homeostatic cytokines IL‐7 and IL‐15 [3, 4, 5, 6, 7]. In particular, CD4^+^ memory T‐cells express the IL‐7Rα chain and require IL‐7, while CD8 memory T‐cells may downregulate IL‐7Rα but express high levels of the IL‐2/15Rβ chain and require IL‐15. However, IL‐15 also contributes to the antigen‐independent maintenance of anti‐viral Th1 memory cells, together with IL‐7 [4]. Moreover, CD8^+^ T‐cells central and effector memory subsets occupy IL‐7‐ and IL‐15‐dependent niches, respectively [6]. In humans, CD4^+^ and CD8^+^ memory T‐cells proliferate in a TCR‐independent manner in response to IL‐7 and IL‐15, suggesting a conserved role for memory T‐cell homeostasis [8, 9]. IL‐7 and IL‐15 are also available in the bone marrow, where they were shown to co‐localize with CD4^+^ and CD8^+^ memory T‐cells [10, 11]. Notably, T‐cells that express CD69 in the bone marrow display a gene signature of tissue‐resident cells and become quiescent [12, 13]. Whether memory T‐cells are maintained by continuous homeostatic proliferation in the spleen or as quiescent cells in the bone marrow is debated [14]. These two possibilities are, however, not mutually exclusive [15]. Immunological memory may also comprise regulatory T‐cells (Tregs) [16]. FOXP3^+^Tregs, the best‐characterized Treg subset, have down‐regulated the IL‐7Rα chain. They express instead the IL‐2Rα chain (CD25) and their functions critically depend on IL‐2 [17, 18]. FOXP3^+^Tregs are present in the bone marrow [13], suggesting that the bone marrow might also play a role in their maintenance. Also FOXP3^−^Tregs may limit excessive and potentially dangerous immune responses. Namely, adaptive Tregs that produce the anti‐inflammatory cytokine IL‐10, often termed type 1 regulatory T‐cells (Tr1), could play an important role [19]. Very little is known about the antigen‐independent maintenance of Tr1‐cells by cytokines. The proliferation of cloned Tr1‐cells is induced by IL‐15 in vitro [20], but T‐cell clones are generated in long‐term cultures with high doses of IL‐2, a condition that is likely to induce and/or select T‐cells that express high levels of the IL‐2/15Rβ chain [20]. Moreover, Tr1‐cells are highly heterogeneous and molecularly not well‐defined, and IL‐10‐producing helper T‐cells may thus be misclassified as Tr1‐cells [21]. We and others showed, however, that a population of Tr1‐like cells expressing the transcription factor EOMESODERMIN (EOMES) [22, 23] displays a unique and highly characteristic gene expression signature, allowing their unbiased identification in humans and mice [22, 23, 24, 25, 26, 27]. EOMES induces a cytotoxic differentiation program in lymphocytes and antagonizes alternative fates in CD4^+^ T‐cells. EOMES^+^Tr1‐like cells express GzmK, produce high levels of IFN‐γ and IL‐10, and are involved in several immune‐mediated diseases, including multiple sclerosis [25, 28]. Human CD4^+^EOMES^+^Tr1‐like cells are effector‐like cells that have down‐regulated the IL‐7Rα chain and have a high in vivo turnover rate, presumably because they are chronically activated by persistent antigens [29]. However, if they are short‐lived effector cells are maintained long‐term in a quiescent state in the bone marrow, as proposed for memory T‐cells, is unknown. We, therefore, analyzed here the characteristics of EOMES^+^Tr1‐like cells in the human bone marrow.

## 2 Results and Discussion

### 2.1 FOXP3^+^ and EOMES^+^CD4^+^T‐Cells Are Enriched in the CD69^+^KI67^−^ Fraction of the Human Bone Marrow

**FIGURE 1:**
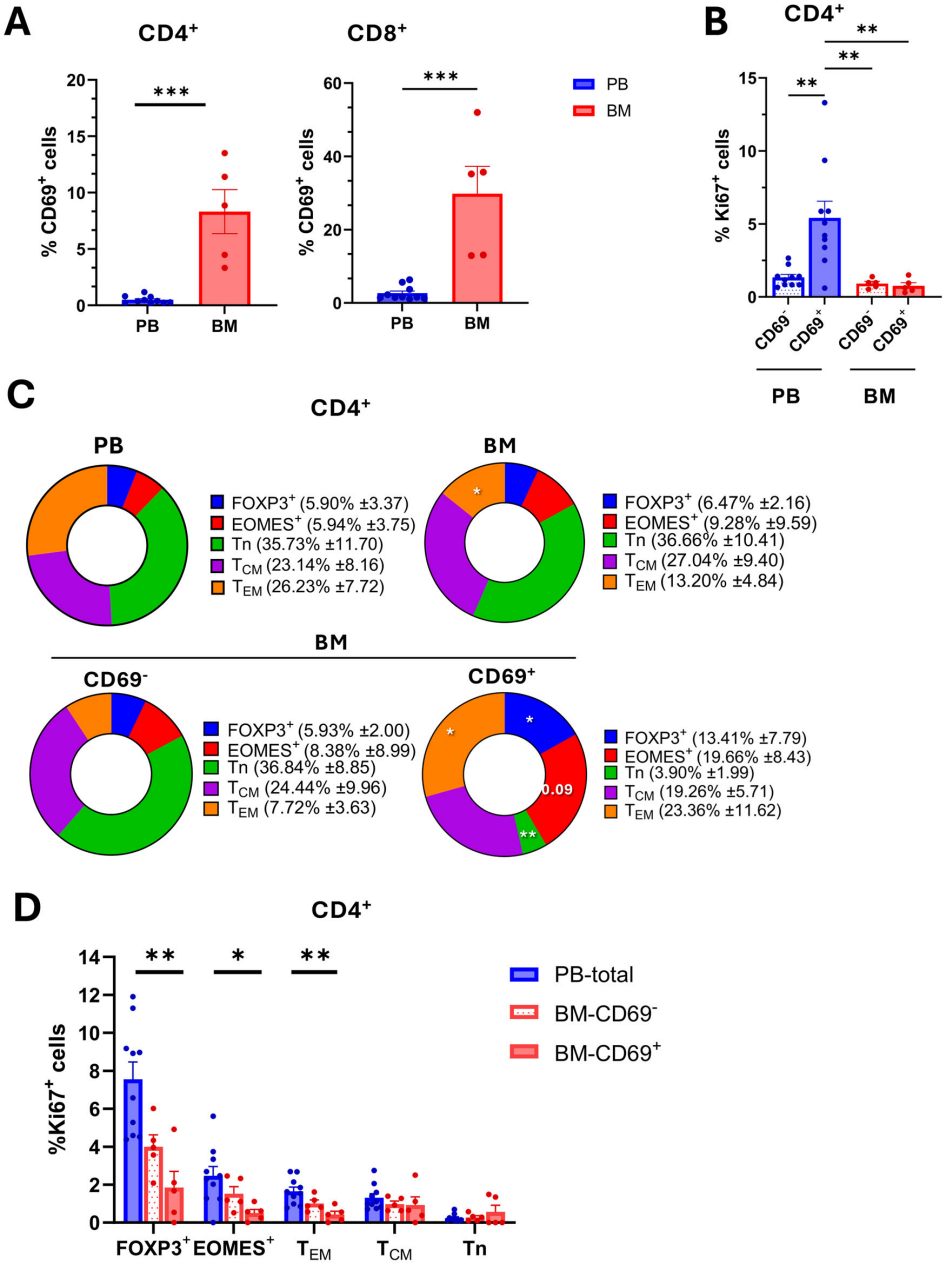
EOMES^+^ and FOXP3^+^CD4^+^T‐cells in the bone marrow upregulate CD69 and become quiescent. (A) Percentages of CD69^+^ cells among CD4^+^ and CD8^+^T‐cells in the blood (PB, *n* = 11) and in the bone marrow (BM, *n* = 5). (B) Percentages of Ki67^+^ cells among CD69^+^ and CD69^−^CD4^+^T‐cells in the blood and the bone marrow. (C) Upper pie charts illustrate the contributions of naïve, central memory, and effector memory cells as well as FOXP3^+^ and EOMES^+^ cells to the CD4^+^T‐cell compartment in the blood and the bone marrow. Lower pie charts show the contributions of the same subsets to the CD69^+^ and CD69^−^ compartments in the bone marrow. Statistical tendencies (*p*‐values >0.05) are reported as numbers in the pie charts. (D) Ki67 expression in the CD4^+^T‐cell subsets analyzed in (C) in peripheral blood and in the CD69^+^ and CD69^−^ fractions in the bone marrow.

We analyzed T‐cells in two cohorts of healthy human donors in the bone marrow and peripheral blood by flow cytometry (Figure S1, Table S2A). CD4^+^ and CD8^+^T‐cells in peripheral blood expressed only low levels of CD69 (Figure 1A). In the bone marrow, significantly higher fractions of T‐cells expressed CD69, as reported previously [12]. Conversely, comparable fractions of CD3^−^ lymphocytes in the blood and the bone marrow expressed CD69 (Figure S2A, B), suggesting that the increased CD69 expression in the bone marrow is characteristic of T‐cells. CD69^+^CD4^+^ T‐cells in the blood expressed significantly higher levels of the proliferation marker Ki67 compared with their CD69^−^counterparts (Figure 1B), consistent with the notion that they are activated cells that have recently divided in vivo. Conversely, CD4^+^CD69^+^ T‐cells in the bone marrow expressed only very low levels of Ki67 (Figure 1B), indicating that they were largely resting. Notably, CD8^+^ T‐cells expressed only low levels of Ki67 in all cases (Figure S2C). In marked contrast, a relatively high fraction of CD3^−^CD69^+^ lymphocytes in the bone marrow expressed Ki67 (Figure S2B), suggesting again a characteristic behavior of T‐cells. Overall, these data are consistent with previous reports by Radbruch and colleagues showing that fractions of T‐cells in the bone marrow express CD69 and are largely quiescent [12, 13].

CD4^+^ T‐cells that expressed the transcription factor EOMESODERMIN (EOMES) were enriched in the bone marrow, although this increase did not reach statistical significance (Figure 1C). Conversely, effector memory T‐cells (T_EM_) were significantly reduced, while naïve T‐cells, central memory T‐cells (T_CM_), and FOXP3^+^Tregs were present at similar frequencies. No major changes between CD8^+^ naïve and memory T‐cell subsets in the blood and the bone marrow were observed (Figure S2D). CD69^+^ cells in the bone marrow display a gene signature of tissue‐resident T‐cells cells [12]. We, therefore, refer to CD69^+^T‐cells in the bone marrow as “bone marrow‐resident” cells in this report, although in the accompanying article by Revueltas et al. [30] evidence is provided that surface CD69‐ memory T cells of the bone marrow are residents as well, expressing CD69 intracellularly, as well as S1PR1 and the transcription factor Krüppel‐like factor 2 (KLF2).

In the CD4^+^T‐cell compartment, naïve T‐cells were strongly and significantly reduced among CD69^+^, bone marrow‐resident cells, whereas T_EM_ were significantly enriched (Figure 1C). This was also observed in the CD8^+^T‐cell compartment (Figure S2D). Furthermore, FOXP3^+^Tregs and EOMES^+^T‐cells cells were strongly enriched among bone marrow‐resident CD4^+^T‐cells (Figure 1C), but the percentages of EOMES^+^CD4^+^T‐cells in individual donors were highly variable, and the increases failed to reach statistical significance (*p* = 0.09). Notably, in the accompanying article by Revueltas et al. [30], the authors performed CITE sequencing and identified five major clusters of CD4^+^CD69^+^T‐cells in the bone marrow. Two of these clusters (2 and 4) expressed cytotoxic molecules, and a third cluster (cluster 6) expressed FOXP3, consistent with an enrichment of cytotoxic and regulatory T‐cells among bone marrow resident CD4^+^T‐cells, and providing single‐cell transcriptome reference data on the cells described here.

FOXP3^+^ and EOMES^+^T‐cells in peripheral blood contained higher frequencies of cells expressing the proliferation marker Ki67 compared with naïve or memory CD4^+^T‐cells (Figure 1D), indicating a more rapid in vivo turnover. Ki67 expression in FOXP3^+^ and CD4^+^EOMES^+^T‐cell subsets was overall lower in the bone marrow (Figure 1B,D), but sizeable fractions of CD69^−^FOXP3^+^ and CD69^−^EOMES^+^CD4^+^T‐cells expressed nevertheless Ki67. Conversely, bone marrow‐resident FOXP3^+^Tregs and EOMES^+^CD4^+^T‐cells expressed significantly lower levels of Ki67 compared with peripheral blood (Figure 1D). A decrease in Ki67 expression was also observed among bone marrow‐resident CD4^+^T_EM_, but not among CD4^+^T_CM_ (Figure 1D)_._ A similar trend was again observed in the CD8^+^T‐cell compartment (Figure S2E). Conversely, naïve CD4^+^ and CD8^+^T‐cells were in all cases largely quiescent.

Overall, these findings indicate that FOXP3^+^Tregs and EOMES^+^CD4^+^T‐cells are enriched among CD69^+^Ki67^−^ cells in the bone marrow of healthy donors, suggesting that regulatory and/or cytotoxic CD4^+^T‐cells could become bone‐marrow‐resident and quiescent.

### 2.2 EOMES^+^GzmK^+^CD4^+^ and CD8^+^T‐Cells, but Not GzmB^+^T‐cells, Are Enriched Among CD69^+^ Bone Marrow‐Resident, Quiescent Cells

**FIGURE 2:**
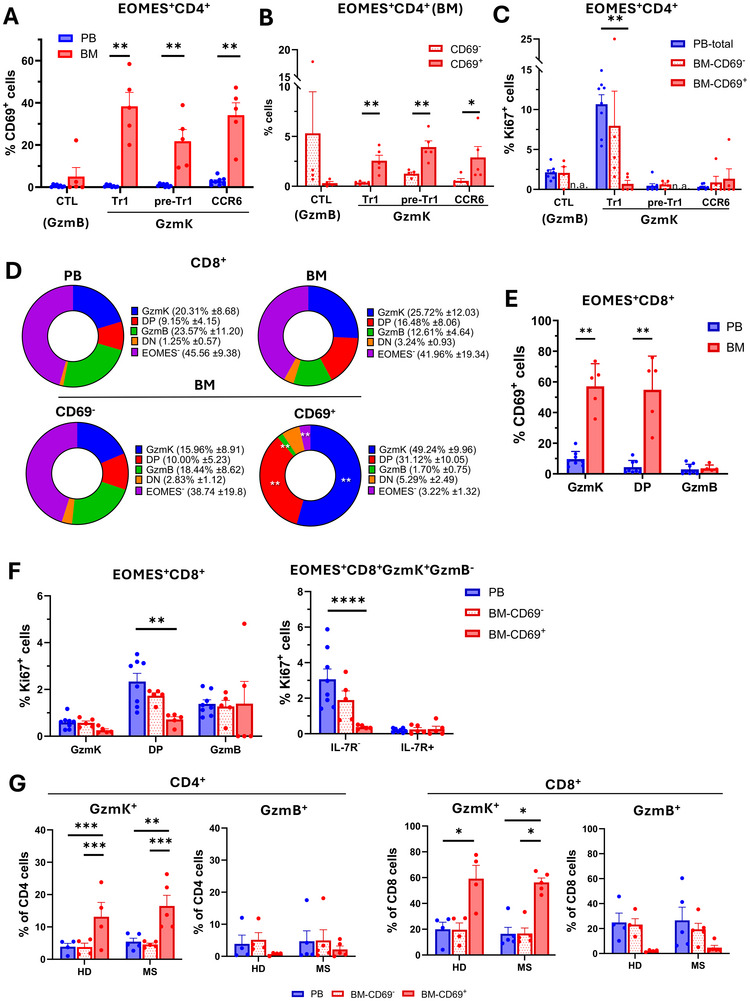
Bone marrow‐resident CD4^+^ and CD8^+^T‐cells are strongly enriched for GzmK‐expressing subsets, whereas GzmB^+^ effector CTL are largely excluded. (A) Percentages of CD69^+^ cells among CD4^+^EOMES^+^T‐cell subsets in the blood and in the bone marrow (BM). (B) Frequencies of CD4^+^EOMES^+^T‐cell subsets among CD69^+^ and CD69^−^CD4^+^T‐cells in the bone marrow. (C) Ki67 expression in CD4^+^ EOMES^+^T‐cell subsets in peripheral blood and the CD69^+^ and CD69^−^ fractions in the bone marrow. (D) The upper pie charts show the contributions of EOMES^+^ subsets classified according to the expression of GzmK and/or GzmB, as well as EOMES^−^ cells, to the CD8^+^T‐cell compartments in the blood and the bone marrow. Lower pie charts show the contributions of the same subsets to the CD69^+^ and CD69^−^ compartments in the bone marrow. (E) Percentages of CD69^+^ cells among CD8^+^EOMES^+^T‐cell subsets in the blood and the bone marrow. (F) Ki67 expression in CD8^+^EOMES^+^T‐cell subsets in peripheral blood and in the CD69^+^ and CD69^−^ fractions in the bone marrow. The left panel shows CD8^+^EOMES^+^T‐cell subsets expressing GzmK and/or GzmB (DP: double‐positive: GzmK^+^GzmB^+^), the right panel CD8^+^EOMES^+^GzmK^+^GzmB^−^T‐cell cells stratified according to IL‐7Rα expression. (G) Percentages of GzmK^+^ and GzmB^+^T‐cell subsets in paired samples of peripheral blood (PB), and in the CD69^+^ and CD69^−^ fractions of the bone marrow (BM) in four healthy donors (HD) and in five multiple sclerosis patients (MS). Upper histograms show CD4^+^T‐cells and lower histograms CD8^+^T‐cells.

Human CD4^+^EOMES^+^T‐cells are heterogeneous and contain terminally differentiated cytotoxic effector cells that express GzmB (“CTL”), and IL‐10 producing regulatory T‐cells (Tr1‐like) that express GzmK [23, 25]. In addition, they also contain proinflammatory, CCR6^+^GzmK^+^ “Th1/17”‐cells and CCR6^−^GzmK^+^Th1‐cells (Figure S1). The latter can be distinguished from Tr1‐like cells by IL‐7Rα expression and are called here “pre‐Tr1” since they were predicted to contain precommitted precursors of Tr1‐cells [31]. Strikingly, all CD4^+^Gzmk^+^ subsets contained high frequencies of CD69^+^ cells in the bone marrow, while CD4^+^GzmB^+^CTL were largely CD69^−^ (Figure 2A). Consequently, GzmK^+^CD4^+^T‐cells were strongly enriched among CD69^+^ bone marrow‐resident cells, whereas CD4^+^CTL were largely excluded (Figure 2B). Notably, CD4^+^CTL were, in contrast, not excluded from CD69^+^CD4^+^T‐cells in the blood (Figure S3A). In the accompanying article by Revueltas et al. [30], the CD4^+^T‐cell Cluster 4 (“T‐PD‐1”), which was enriched in the CD69^+^ fraction of the bone marrow, expressed genes characteristic of EOMES^+^Tr1‐like cells. Moreover, cluster 4 expressed higher levels of GZMK but lower levels of GZMB in the CD69^+^ fraction compared with the CD69^‐^ fraction of the bone marrow. These findings suggest that the enrichment of CD4^+^GZMK^+^ cells and the exclusion of GZMB^+^CD4^+^T‐cells from the bone marrow‐resident, CD69^+^ fraction could also be detected in an unbiased manner by CITE‐seq in unrelated individuals.

EOMES^+^GzmK^+^Tr1‐like cells had high in vivo turnover rates in the blood (Figure 2C), as reported previously [29]. This was also true for CD69^−^Tr1‐like cells in the bone marrow. In marked contrast, CD69^+^, bone marrow‐resident Tr1‐cells were largely quiescent (Figure 2C). Finally, pre‐Tr1 and GzmK^+^CCR6^+^Th1/17‐cells expressed in all cases only low levels of Ki67, while GzmB^+^CD4^+^CTL had intermediate proliferation rates (Figure 2C). Since CD69^+^CD4^+^CTL were hardly detectable in the bone marrow, their Ki67 expression could not be analyzed.

We next investigated if EOMES^+^CD4^+^ and CD8^+^T‐cell subsets in the bone marrow showed similar behaviors. CD8^+^T‐cells expressed high levels of EOMES both in the blood and the bone marrow (Figure S3B). EOMES expression was as expected restricted to CD8^+^ memory cells (Figure S3C). Among the latter, EOMES expression was higher in CD8^+^T_EM_ and T_EMRA_ compared with T_CM_. Moreover, these CD8^+^ memory subsets expressed different patterns of GzmK and GzmB. Thus, in the blood EOMES^+^T_CM_ and T_EM_ expressed mainly GzmK, whereas T_EMRA_ expressed predominantly GzmB, corresponding to cells of cluster 2 and cluster 3 in the accompanying paper of Revueltas et al. [30]. Since GzmK and GzmB are associated with CD8^+^ memory and effector cells, these findings are consistent with the notion that T_EMRA_ contains terminally differentiated effector cells [8, 32, 33]. In the bone marrow, there was, however, a significant shift from GzmB to GzmK expression among T_EMRA_ (Figure S3C), suggesting that they were more memory‐like in the bone marrow.

In order to compare EOMES^+^CD8^+^ with the previously analyzed EOMES^+^CD4^+^T‐cell subsets (Figure 2A–C), we next analyzed CD8^+^EOMES^+^T‐cells according to GzmB and GzmK expression (Figure 2D). We detected a moderate shift from GzmB to GzmK expression among total CD8^+^T‐cells in the bone marrow. Moreover, among CD69^+^, bone marrow‐resident cells, EOMES and GzmK co‐expressing CD8^+^T‐cells were strongly and significantly enriched (Figure 2D). Consequently, a major fraction of bone marrow‐resident CD8^+^T‐cells expressed exclusively GzmK, but also GzmK and GzmB double‐positive (“DP”) cells contributed significantly. Conversely, CD8^+^T‐cells cells that lacked EOMES or GzmK expression were largely excluded from the bone marrow‐resident fraction. As observed among CD4^+^EOMES^+^T‐cells, CD69 expression in the bone marrow was hardly detectable on CD8^+^GzmB^+^GzmK^−^ cells, whereas in mean >50% of cells in CD8^+^GzmK^+^EOMES^+^T‐cell subsets expressed CD69 (Figure 2A,E). Finally, Ki67 expression analysis unveiled that the bone marrow‐resident EOMES^+^GzmK^+^CD8^+^T‐cells expressed significantly lower levels of Ki67 compared with the blood (Figure 2F). Conversely, GzmB^+^GzmK^−^ effector cells showed similar proliferation rates in the blood and the CD69^+^ fraction of the bone marrow. Notably, the increased proliferation rates of CD8^+^EOMES^+^GzmK^+^ T‐cells in the blood were due to cells that had down‐regulated IL‐7Rα expression (Figure 2F; Figure S3D). Consequently, CD8^+^T‐cells that displayed the same phenotype as CD4^+^EOMES^+^Tr1‐like cells, that is, EOMES^+^GzmK^+^IL‐7R^lo^, proliferated in the blood but became tissue‐resident and quiescent in the bone marrow (Figure 2F).

In order to corroborate our findings in independent cohorts, we next analyzed paired blood and bone marrow samples from five multiple sclerosis patients and four healthy control donors (Figure 2G). Notably, the latter were distinct from the previously analyzed healthy donors (Figures 1 and 2A–F). In both MS patients and healthy control donors, we confirmed that CD4^+^ and CD8^+^T‐cells expressing GzmK were enriched among CD69^+^, bone marrow‐resident cells, while GzmB^+^CTL were excluded (Figure 2G).

In conclusion, these findings suggest that EOMES^+^GzmK^+^Tr1‐like cells home to the bone marrow, upregulate CD69, and become quiescent. It is thus tempting to speculate that they could be maintained long‐term in the bone marrow like memory cells. Conversely, GzmB^+^ effector CTL does not acquire features of bone marrow‐resident cells and may thus rather represent short‐lived effector T‐cells. The strikingly different capacities of EOMES^+^GzmK^+^ and GzmB^+^T‐cell subsets to become bone marrow‐resident and quiescent were observed both in the CD4^+^ and CD8^+^T‐cell compartments, and under steady‐state and autoimmune conditions. Since GzmK^+^ and GzmB^+^ T‐cell subsets express different levels of T‐bet [23], their strikingly different behavior in the bone marrow might be explained by the concept that EOMES and T‐bet promote features of respectively memory and effector cells in CD8^+^T‐cells in mice [34].

### 2.3 EOMES Induces IL‐2/15Rβ in Tr1‐Like Cells, and IL‐15 Rescues EOMES^+^Tr1‐Like Cells From Death by Neglect

**FIGURE 3:**
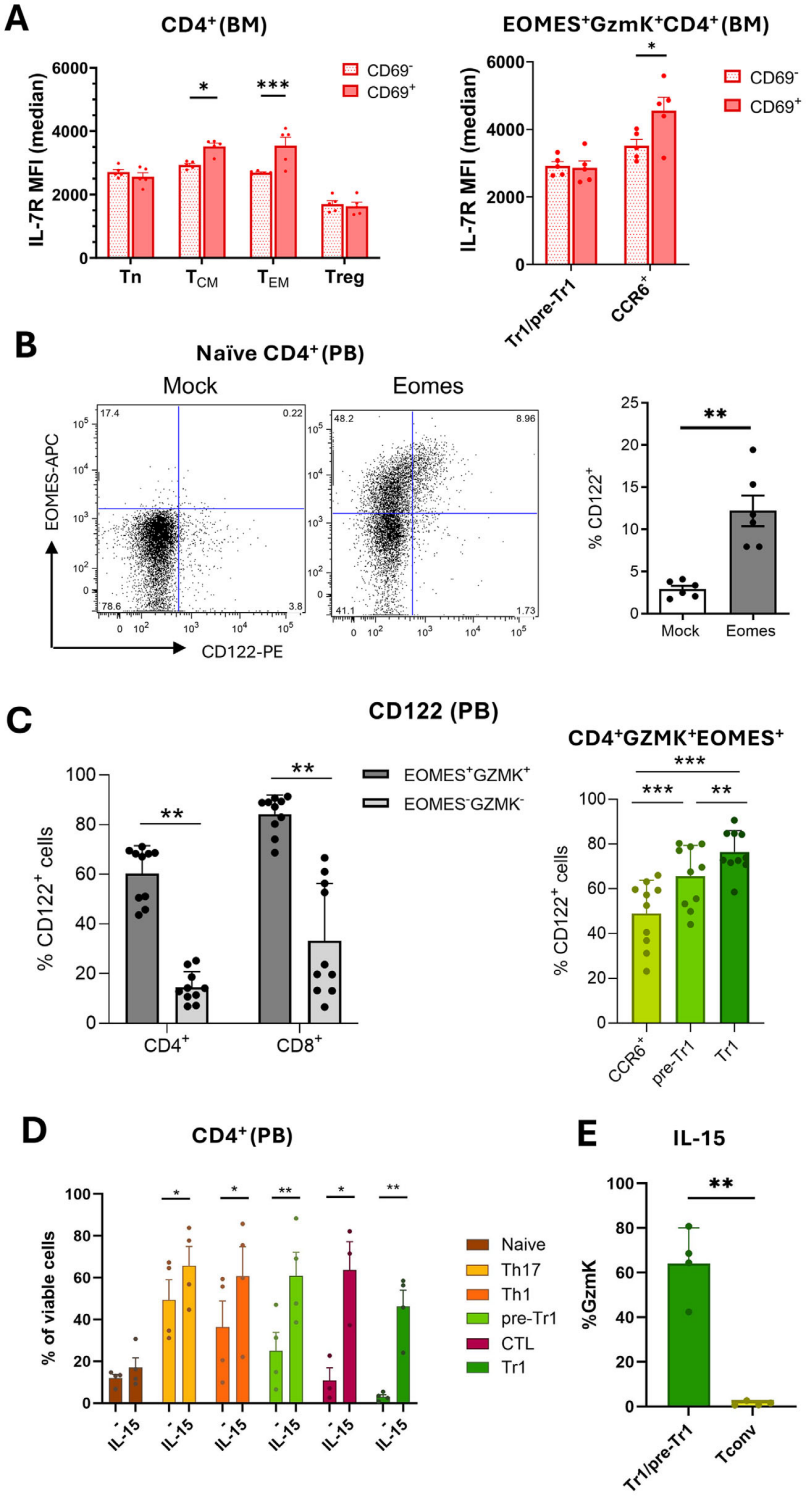
IL‐7R^lo^EOMES^+^Tr1‐like cells express high levels of IL‐2/15Rβ and are rescued from death by neglect by IL‐15. (A) IL‐7Rα expression levels on CD4^+^T‐cell subsets in the CD69^+^ and CD69^−^ compartments of the bone marrow. The upper panel shows naïve, central, and effector memory cells as well as FOXP3^+^Treg and the lower panel shows CD4^+^EOMES^+^GzmK^+^ subsets. Representative histogram overlays are shown in Figure S4A. (B) Naive CD4^+^T‐cells were activated with anti‐CD3 and anti‐CD28 antibodies, IL‐2 and IL‐12, and transduced with lentiviral vectors coding for GFP (Mock) or for GFP and Eomesodermin (Eomes). Left: Representative Dot plots of cells analyzed for EOMES and CD122 (IL‐2/15Rβ) expression. Mean percentage of CD122 expression (*n* = 6). (C) Percentages of CD122^+^ cells in the indicated T‐cell subsets. The gating strategy is shown in Figure S5. (D) CD4^+^T‐cell subsets were enriched by cell sorting according to the gating strategy reported in Figure S6. They were cultured for 4 days in the absence or presence of IL‐15, and the mean percentage of viable cells was reported (*n* = 4). (E) Pre‐Tr1/Tr1‐cell‐enriched populations and CD4^+^CCR5^−^CD27^+^ Tconv control cells were cultured for 4 days with IL‐15, and GzmK expression of viable cells was analyzed.

Antigen‐independent proliferation and survival of memory T‐cells in the bone marrow are systemically controlled by the homeostatic cytokines IL‐7 and IL‐15 [3–6, 8, 9]. T‐cell responsiveness to these cytokines, which both signal via the common γ‐chain, requires the expression of specific cytokine receptor subunits, namely IL‐7Rα and IL‐2/15Rβ [35]. Human blood CD4^+^ memory T‐cells express IL‐7Rα, consistent with the concept that their antigen‐independent survival depends on IL‐7 [7]. FOXP3^+^Tregs [17] and EOMES^+^Tr1‐like cells [29] have, in contrast, a low IL‐7Rα expression, indicating a low responsiveness to IL‐7 [17, 29]. CD4^+^ central memory T‐cells expressed IL‐7Rα also in the bone marrow (Figure 3A; Figure S4A). Interestingly, bone marrow‐resident CD4^+^T_CM_ and T_EM_ subsets expressed significantly higher levels of IL‐7Rα compared with their CD69^−^ counterparts. This increase of IL‐7Rα expression was not observed in CD69^+^ naïve T‐cells and was most prominent for T_EM_ (Figure 3A; Figure S4A). The selective increase of IL‐7Rα expression on bone marrow‐resident memory cells is consistent with the proposed key role of IL‐7 for CD4 memory maintenance in the bone marrow [36] and may reflect IL‐7R signaling [37]. FOXP3^+^Tregs and EOMES^+^GzmK^+^CCR6^−^Tr1/pre‐Tr1‐like cells in the bone marrow expressed, in contrast, in all cases relatively low levels of IL‐7Rα (Figure 3A; Figure S4A), suggesting that the survival of regulatory T‐cells in the bone marrow depends on other common γ‐cytokines. Interestingly, CD4^+^CCR6^+^GzmK^+^Th1/17‐cells expressed the highest levels of IL‐7Rα of all CD4^+^ T‐cell subsets and showed a similar behavior as CD4^+^ memory T‐cells, since IL‐7Rα expression levels were further increased among bone marrow‐resident cells (Figure 3A; Figure S4A). In the CD8^+^T‐cell compartment, bone marrow‐resident T_CM_ had an increased expression of IL‐7Rα (Figure S4B). Conversely, bone marrow‐resident CD8^+^GzmK^+^T‐cells expressed significantly lower levels of IL‐7Rα, suggesting again a role for other survival‐promoting cytokines like IL‐15. Consistently, IL‐15 was shown to downregulate IL‐7Rα expression on CD8^+^ memory T‐cells in the murine bone marrow in vivo [37].

FOXP3^+^Tregs express IL‐2Rα and their function is controlled by IL‐2 [17, 18]. Conversely, the cytokines that control EOMES^+^Tr1‐like cells are unknown. EOMES induces IL‐2/15Rβ‐chain (CD122) expression and memory features in murine CD8^+^T‐cells [34]. Forced expression of EOMES human blood naïve CD4^+^T‐cells activated with anti‐CD3/28 antibodies in the presence of IL‐12 significantly increased CD122 expression (Figure 3B), suggesting that the induction of IL‐15 responsiveness by EOMES is conserved in different T‐cell compartments and between different species. Among human blood T‐cells ex vivo, CD122 was as expected expressed on major fractions of CD8^+^T‐cells, but only on minor fractions of CD4^+^T‐cells (Figure S5A,C). However, CD122 was expressed at high levels on FOXP3^+^Tregs (Figure S5B,D), consistent with the key role of IL‐2 in Treg biology. Moreover, CD122 was also expressed on major fractions of EOMES^+^GzmK^+^CD4^+^ and CD8^+^T‐cells (Figure 3C). Among EOMES^+^GzmK^+^CD4^+^T‐cells, Tr1‐like cells expressed the highest levels of CD122 and CCR6^+^GzmK^+^T‐cells the lowest (Figure 3C).

To address whether the IL‐15R on CD4^+^T‐cell subsets was functional, we investigated the pro‐survival effects of IL‐15 on FACS‐purified T‐cell subsets (Table S2B). We sorted CD4^+^T‐cell populations that were enriched for pre‐Tr1, Tr1‐like cells, and CTL from human blood according to surface markers (Figure S6A,B). We also isolated CD4^+^ naïve T‐cells, which lack CD122 expression, and CCR5^−^Th1 and Th17 memory cells, which express low levels of CD122, as additional controls. CD122 expression levels in CTL, Tr1, pre‐Tr1, and Th1 memory cells were controlled by RT‐PCR (Figure S6C) and confirmed high CD122 expression of the Tr1‐enriched populations. Major fractions of the FACS‐enriched CD4^+^CTL, and approximately half of the cells in the pre‐Tr1 and Tr1‐cell‐enriched populations, expressed EOMES and Granzymes alone or in combinations (Figure S6B). As reported previously, CTL expressed mainly GzmB, whereas Tr1‐ and pre‐Tr1‐cells expressed predominantly GzmK [23]. Conversely, Th1, Th17 memory, and naïve T‐cells were largely devoid of cells expressing these cytotoxicity‐associated molecules. In vitro cultures in the absence of survival‐inducing cytokines for 4 days unveiled strikingly different capacities to resist death by neglect of the purified T‐cell populations (Figure 3D). Thus, while nearly half of Th17 and Th1 memory cells survived, naïve cells and also virtually all cells in the Tr1‐ and CTL‐enriched populations died. Pre‐Tr1‐enriched populations showed an intermediate resistance to death by neglect. Importantly, recombinant IL‐15 had a very strong pro‐survival effect on the populations enriched for EOMES^+^Tr1‐like cells and CD4^+^CTL (Figure 3D). In marked contrast, naïve T‐cell survival was not rescued by IL‐15. Finally, IL‐15 had a weak but significant prosurvival effect on Th17 and Th1 memory cells, and populations enriched for pre‐Tr1‐cells showed again an intermediate behavior (Figure 3D). IL‐15 largely failed to induce proliferation under these conditions, as evidenced by the overall low levels of Ki67 in IL‐15‐supplemented cultures (Figure S6D).

To exclude that IL‐15 promoted the survival of contaminating EOMES^−^GZM^−^ effector cells rather than of EOMES^+^Tr1‐like cells, we then enriched CD4^+^T‐cell populations containing Tr1‐ and pre‐Tr1‐cells also according to PD1 expression [23] (Figure S7A, Table S2B). The large majority of CD4^+^T‐cells purified with this more stringent strategy expressed EOMES and/or GzmK ex vivo (Figure S7B), consistent with previous results [23]. Moreover, the majority of cells in these Tr1/pre‐Tr1‐enriched populations that survived in the presence of IL‐15 (Figure S7C) expressed GzmK (Figure 3E), consistent with the notion that IL‐15 promotes the survival of pre‐Tr1 and Tr1‐like cells. Notably, conventional CD4^+^CD25^−^ control T‐cells, which were sorted as CCR5^−^CD27^+^ and therefore lacked EOMES and GZMK expression ex vivo (Figure S7A,B, Table S2B), remained GzmK‐negative under the same conditions (Figure 3E; Figure S7C).

In summary, EOMES promotes IL‐2/15Rβ expression in CD4^+^T‐cells, leading to IL‐15 responsiveness and IL‐15‐dependent rescue from death by neglect [38]. Data from mouse models suggest further that EOMES [39] or IL‐15 in the bone marrow [37] may also contribute to IL‐7Rα downregulation. Other possible factors are chronic, low‐quality TCR stimulation [38, 40] and IL‐27 [23]. In any case, our findings suggest that developing EOMES^+^Tr1‐like cells progressively switch cytokine receptor expression and consequently switch from IL‐7 to IL‐15 responsiveness. The reciprocal cytokine receptor expression patterns suggest furthermore that EOMES^+^IL‐7Rα^lo^Tr1‐like cells and CD4^+^IL‐7Rα^hi^ memory T‐cells compete for largely distinct, that is, IL‐7 and IL‐15‐dependent, survival niches systemically and also in the bone marrow [6, 10, 11]. Finally, the intermediate responsiveness of IL‐7Rα^+^Th1‐cells and pre‐Tr1‐cells to IL‐15 is consistent with the concept that Th1‐cells are maintained jointly by IL‐7 and IL‐15 [4, 41].

### 2.4 Data Limitations and Perspectives

Experimental studies on the human immune system have intrinsic limitations that also apply to this study. The enrichment of CD69^+^, resting EOMES^+^Tr1‐like cells in the bone marrow suggests, but does not demonstrate, that they are maintained long‐term in this primary lymphoid organ. This needs to be formally demonstrated in appropriate mouse models in the future. Finally, given the limited number of T‐cells in the bone marrow samples, we were unable to perform the survival assays with bone marrow‐resident cells.

### 2.5 Concluding Remarks

The bone marrow offers homeostatic niches for the antigen‐independent maintenance of memory T‐cells. Here we provided evidence that the bone marrow offers also survival niches for regulatory T‐cells, including both FOXP3^+^Tregs and EOMES^+^Tr1‐like cells that are likely to have different cytokine requirements [16]. EOMES induces a high IL‐15 responsiveness in Tr1‐like cells that distinguishes them from IL‐7‐responsive CD4^+^ memory T‐cells, suggesting that CD4^+^ memory and regulatory T‐cells compete for different survival niches. Bone marrow‐resident FOXP3^+^ regulatory T‐cells play a role in the maintenance of hematopoietic stem cells [42], and a future challenge is to understand if bone‐marrow‐resident EOMES^+^Tr1‐like cells may play a similar role.

## 3 Materials and Methods

### 3.1 Human Samples

Bone marrow (BM) samples from nine healthy BM donors were collected, and in four cases, peripheral blood mononuclear cells were obtained from the same patients. In addition, peripheral blood mononuclear cells from 11 unrelated healthy donors were collected. BM samples were also collected from five patients with multiple sclerosis (MS) before undergoing autologous hematopoietic stem cell transplantation. The study (OSSMA) was approved Ethics Committee Regione Liguria (Approvazione CER Liguria, n. registro 052/2019) and by the Ethical Committee Milano Area 2 (immunnom/2020, parere 708_2020).

### 3.2 Flow Cytometry

T‐cells were analyzed directly ex vivo on a FACS Symphony machine according to the gating strategy in Figure S1. Cells were fixed and permeabilized with eBioscience Foxp3/transcription factor staining buffer set. The used antibodies are listed in Table S1. The markers used to identify T‐cell subsets are indicated in Table S2.

### 3.3 Statistical Analysis

Statistical analysis was performed after verifying the normal distribution of the data with the Shapiro–Wilk normality test. For normally distributed data, a *t*‐test (Figure 3B [unpaired], 3D, 3E, Figures S2D [unpaired], S5C, S5D, S7C [unpaired]), one‐way ANOVA (Figures 1, 2 [left], 3C [right], S6C), or two‐way ANOVA (Figures 2G [top], 3A, S3A, S4B) was applied. For non‐normally distributed data, either the Mann–Whitney test (Figures 1, 2, 2A,B,D,E; Figures S2B, S3C) or the Kruskal–Wallis test (Figures 1, 2 [right], 2G [bottom]; Figure S3D) or Wilcoxon matched‐pairs test (Figure 3C [left]) was used. *, **, ***, and **** report statistically significant *p*‐values of ≤0.05, ≤0.001, ≤0.0001, and ≤0.00001, respectively.

### 3.4 Lentivirus‐Mediated EOMES Gene Transfer in Naïve CD4 T Cells

Forced expression of EOMES in primary human CD4^+^T‐cells was performed as previously described [23]. Briefly, FACS‐purified CD4^+^ naive cells (CCR7^+^CD45RA^+^) were activated at a density of 10^5^ cells per well in 96‐well coated with anti‐CD3 (0.1 µg/mL; UCHT1; BD) and anti‐CD28 (6 µg/mL; CD28.2; BD), IL‐2 (20 IU/mL; Novartis), and 10 ng/mL IL‐12. Lentiviral particles were produced according to a standard protocol (System Biosciences User Manual). T cells were simultaneously activated and transduced with either GFP control lentiviral vector or a lentiviral vector encoding the whole length wildtype EOMES at a multiplicity of infection of 10^7^ transducing units/mL. Cells were detached on day 3, transferred to uncoated wells, and cultured in a complete RPMI medium. After 7 days, cells were analyzed by flow cytometry for CD122 induction and EOMES expression.

### 3.5 Survival Assay

CD4^+^ T‐cell populations were purified according to the gating strategy illustrated in Figures S6A or S7A. They were cultured for 4 days in the absence or presence of 10 ng/mL IL‐15. Viable cells were tracked according to live/dead dye (Thermofisher).

## Author Contributions

Nadia Pulvirenti, Chiara Vasco, Stefano Maglie, and Petra Dadova performed and analyzed experiments. Camilla Righetti performed statistical analyses. Chiara Vasco, Petra Dadova, and Camilla Righetti edited the paper. Giacomo Boffa, Alice Laroni, and Tiziana Vigo isolated cells from the bone marrow and edited the paper. Maria Cristina Crosti performed cell sorting, Sergio Abrigani provided critical discussion, Antonio Uccelli provided critical discussion and access to BM samples, and Jens Geginat designed the study, supervised experiments, and wrote the paper.

## Conflicts of Interest

The authors declare no conflicts of interest.

### Peer Review

The peer review history for this article is available at https://publons.com/publon/10.1002/eji.202451644.

## Supporting information



Supporting information

Supporting information

Supporting information

## Data Availability

The data that support the findings of this study are available from the corresponding author upon reasonable request.
