# Supplementary material for: The Human Bone Marrow May Offer an IL‐15‐Dependent Survival Niche for EOMES+ Tr1‐Like Cells
Source: Eur J Immunol. 2025 May 16;55(5):e202451644. doi: 10.1002/eji.202451644 (PMC12082382; doi:10.1002/eji.202451644)
Supplement: Supplementary file 1 — Supporting information [file EJI-55-e202451644-s003.pdf]

sFigure 1: Gating strategy to identify EOMES^{+} and EOMES^{-} T-cell subsets

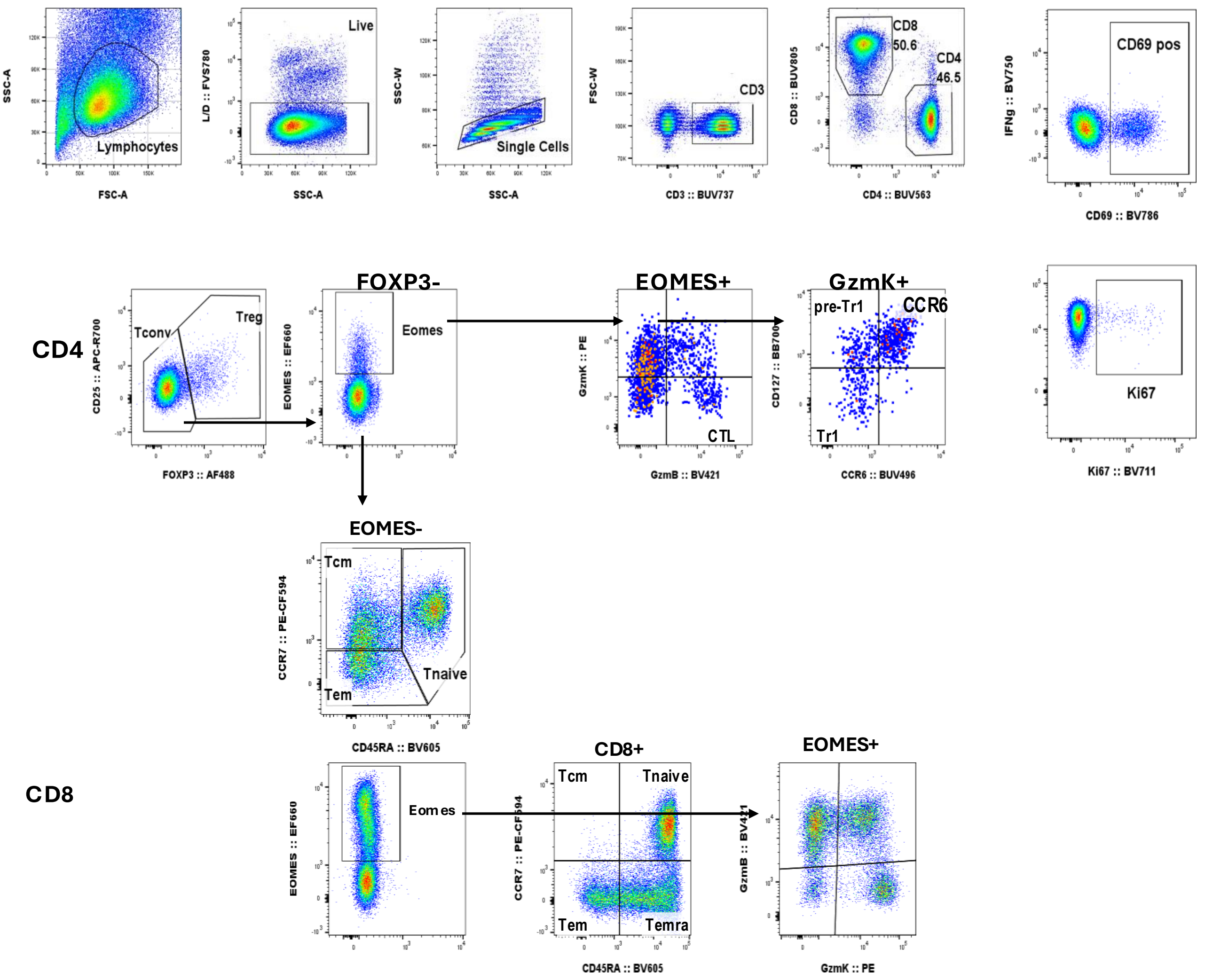

Lymphocytes were gated accordingt to FSC/SSC and dead cells as well as doublets excluded. CD3+T-cells were gated as CD4+ or CD8+ and analysed for CD69 or Ki67 expression. CD4+T-subsets were gated according to FOXP3 expression as Tregs and Tconv. Tconv were analysed for EOMES expression. EOMES^{-} Th cells were analysed for CCR7 and CD45RA expression to identify naïve, central and effector memory cells. CD4+EOMES+T-cells were analysed for GZMK and GZMB expression, and GZMK+ subsets classified according to CCR6 and IL-7R expression as indicated.
CD8+T-cells were analysed for EOMES expression and EOMES+ cells according to GZMK and GZMB Expression. Alternatively, total CD8+T-cells were analysed for CCR7 and CD45RA expression to identify naïve, central memory and effector memory cells, as well as CD45RA+CCR7-TEMRA.

sFigure 2: Proliferation of CD3-negative lymphocytes and of CD8+T-cell subsets in the bone marrow according to CD69 expression

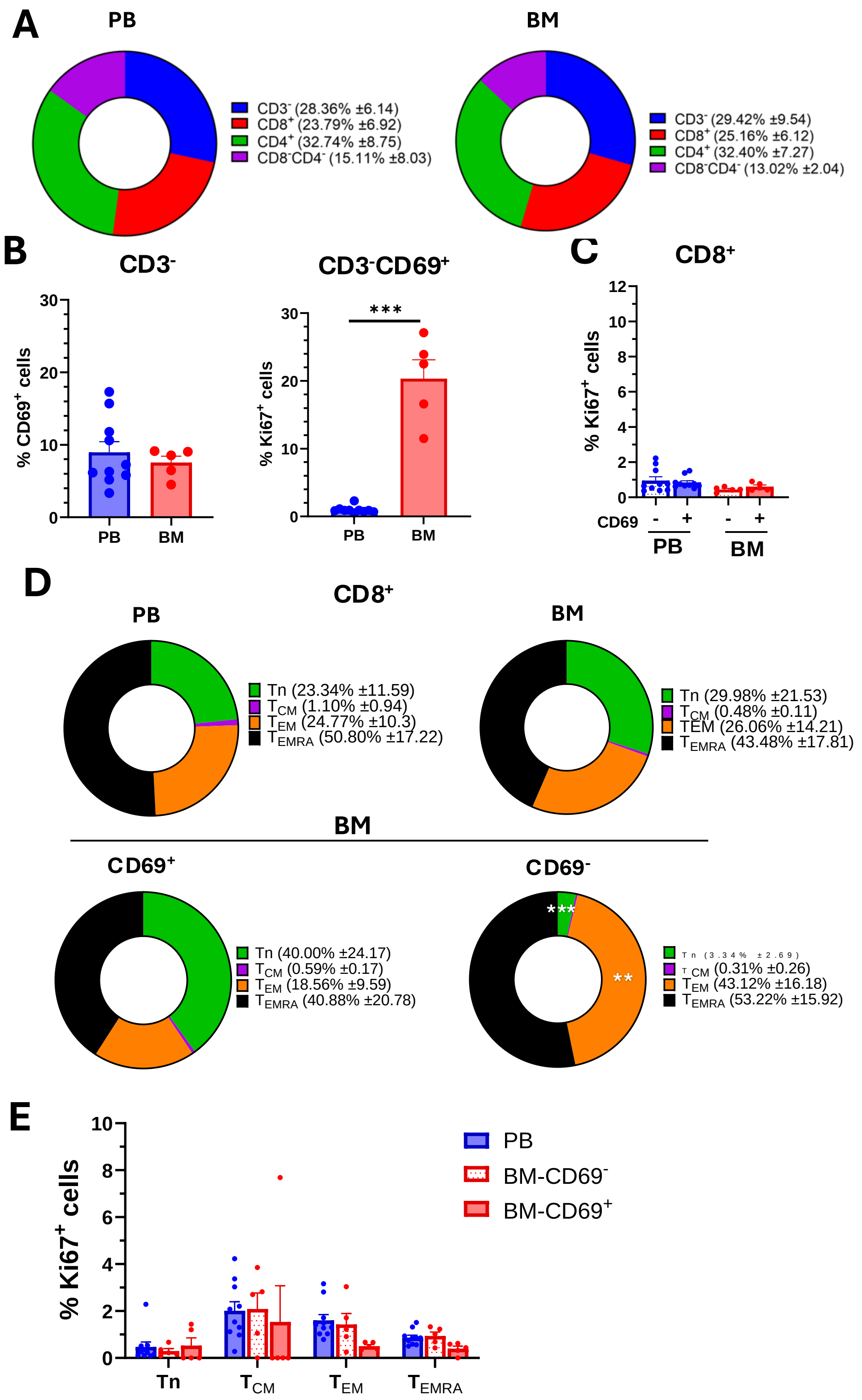

A. Lymphocytes in peripheral blood (PB) and in the bone marrow (BM) were analysed according to the expression of CD3, CD4 and CD8. Pie Charts show the mean percentages of $CD4^+$, $CD8^+$ and double-negative T-cells, as well as non T-cells ($CD3^-$ lymphocytes). **B.** $CD3^-$ lymphocytes in the blood and in the bone marrow were analysed for CD69 (left panel) and Ki67 expression (right panel). **C.** Percentages of $Ki67^+$ cells among $CD69^+$ and $CD69^-CD8^+$T-cells in the blood and in the bone marrow. **D.** Mean contributions of the indicated naïve and memory $CD8^+$T-cell subsets to the $CD69^-$ and $CD69^+$ fractions in peripheral blood and in the bone marrow (upper pie charts), and in the $CD69^-$ and $CD69^+$ fractions of the bone marrow (lower pie charts). **E.** Ki67 expression of naïve and memory $CD8^+$T-cell subsets in peripheral blood and in the $CD69^-$ or $CD69^+$ fractions of the bone marrow.

sFigure 3: Comparison of CD4+CD69+ and CD69-T-cells in the blood and of CD8+T-cells in the bone marrow

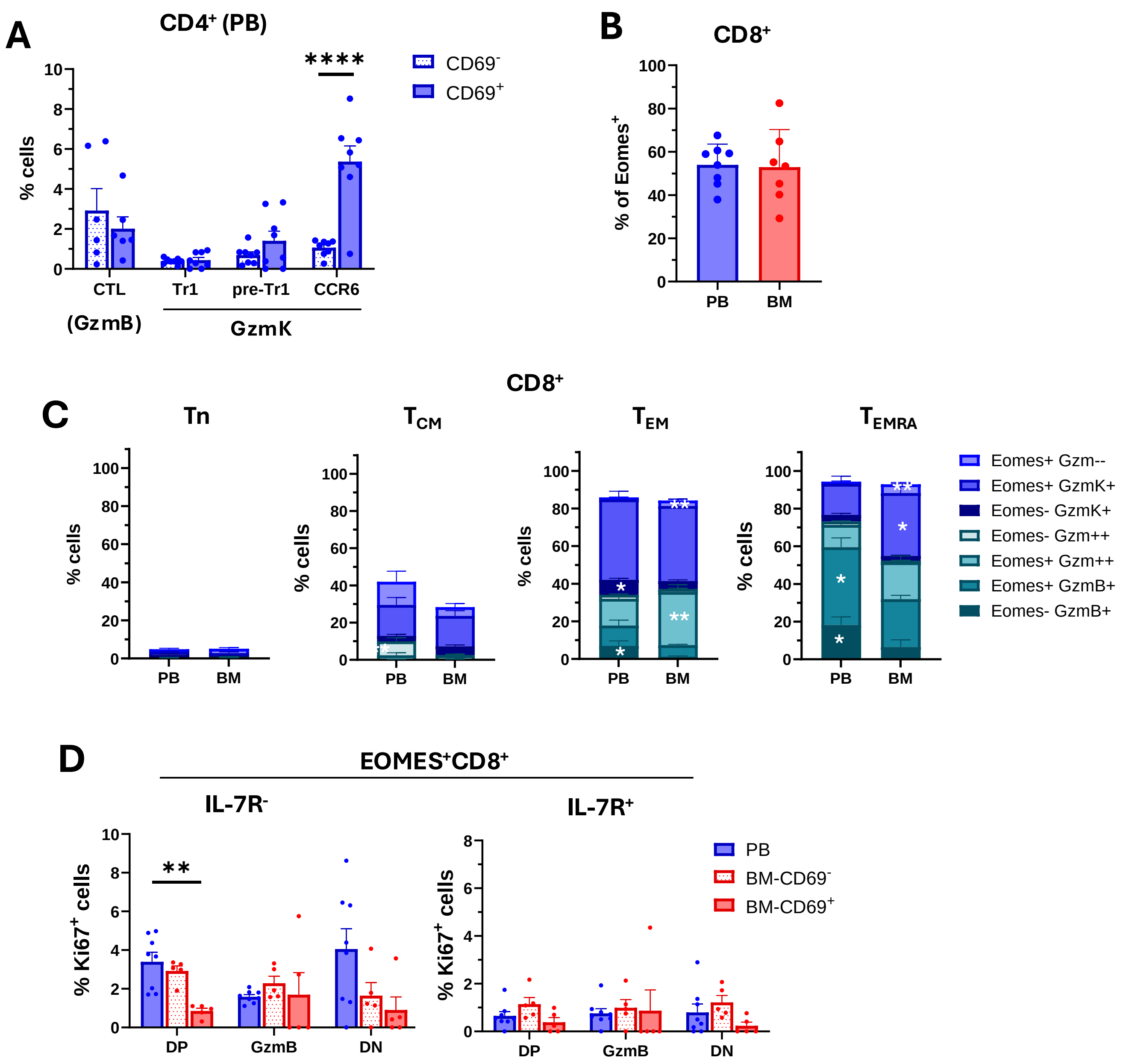

A. Frequencies of CD4$^+$EOMES$^+$T-cell subsets among CD69$^+$ and CD69$^-$CD4$^+$T-cells in peripheral blood (PB). **B**. EOMES expression among CD8$^+$T-cells in peripheral blood and in the bone marrow (BM). **C.** Expression patterns of EOMES, GzmK and GzmB in naïve and memory CD8$^+$T-cell subsets in the blood and in the bone marrow. **D.** Ki67 expression in CD8$^+$EOMES$^+$T-cell subsets according to GzmB and GzmK expression (DP: double-positive, GzmB: GzmB$^+$GzmK$^-$, DN: double-negative) stratified according to IL-7Rα expression in peripheral blood and in the CD69$^+$ and CD69$^-$ fractions in the bone marrow. Ki67 expression of GzmK$^+$GzmB$^-$ cells is shown in Figure 2F.

sFigure 4: IL-7Rα expression in CD4+ and CD8+T-cell subsets

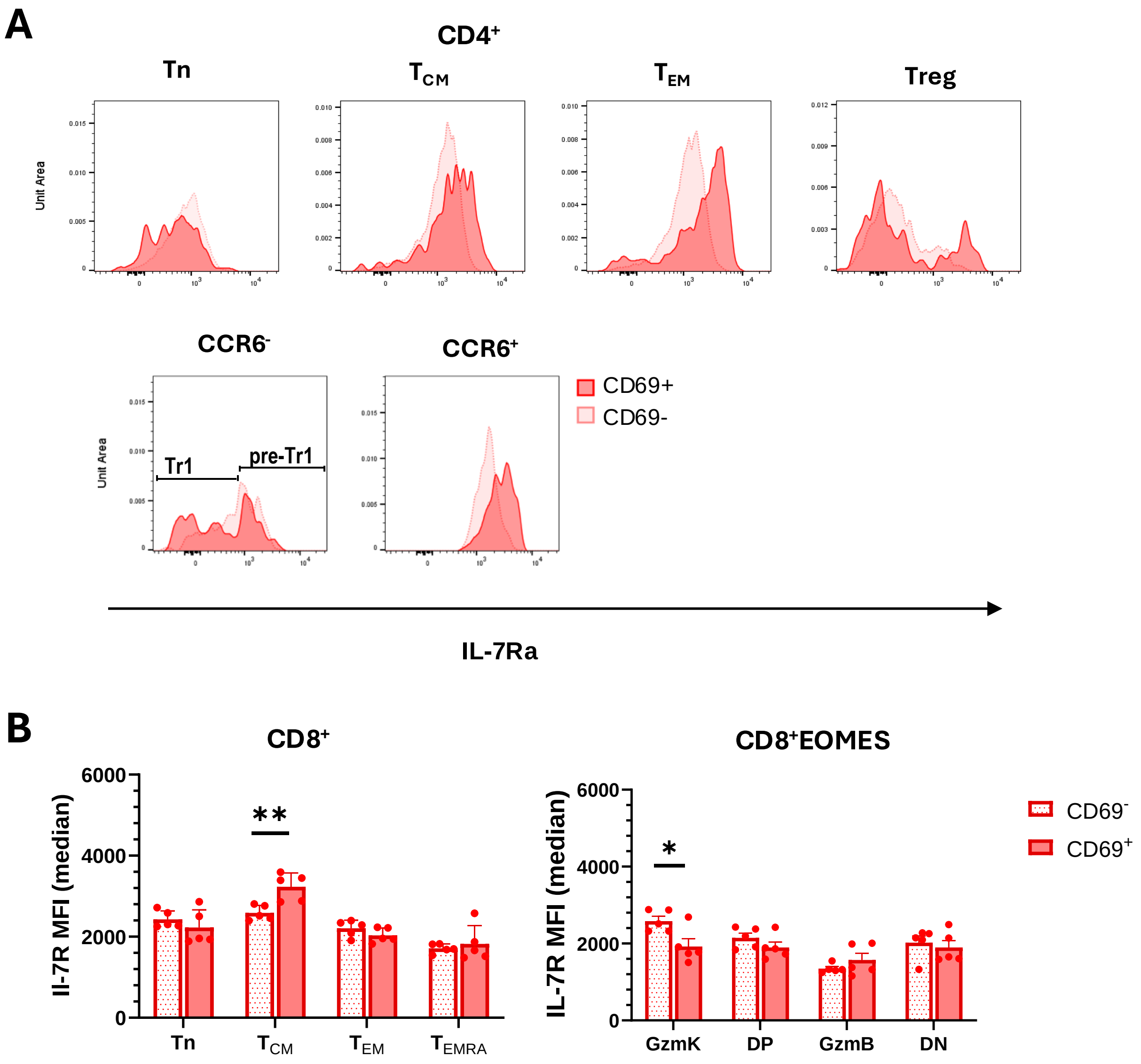

A. Histogram Overlays of IL-7Rα expression on the indicated CD4⁺T-cell subsets in the CD69⁺ and CD69⁻ compartments of the bone marrow. **B.** IL-7Rα expression levels on CD8⁺T-cell subsets in the CD69⁺ and CD69⁻ compartments of the bone marrow. The left panel shows naïve, central and effector memory cells, the right panel CD8⁺EOMES⁺ subsets classified according to GzmK and GzmB expression.

sFigure 5: Gating strategy to analyse CD122 expression on T-cell subsets

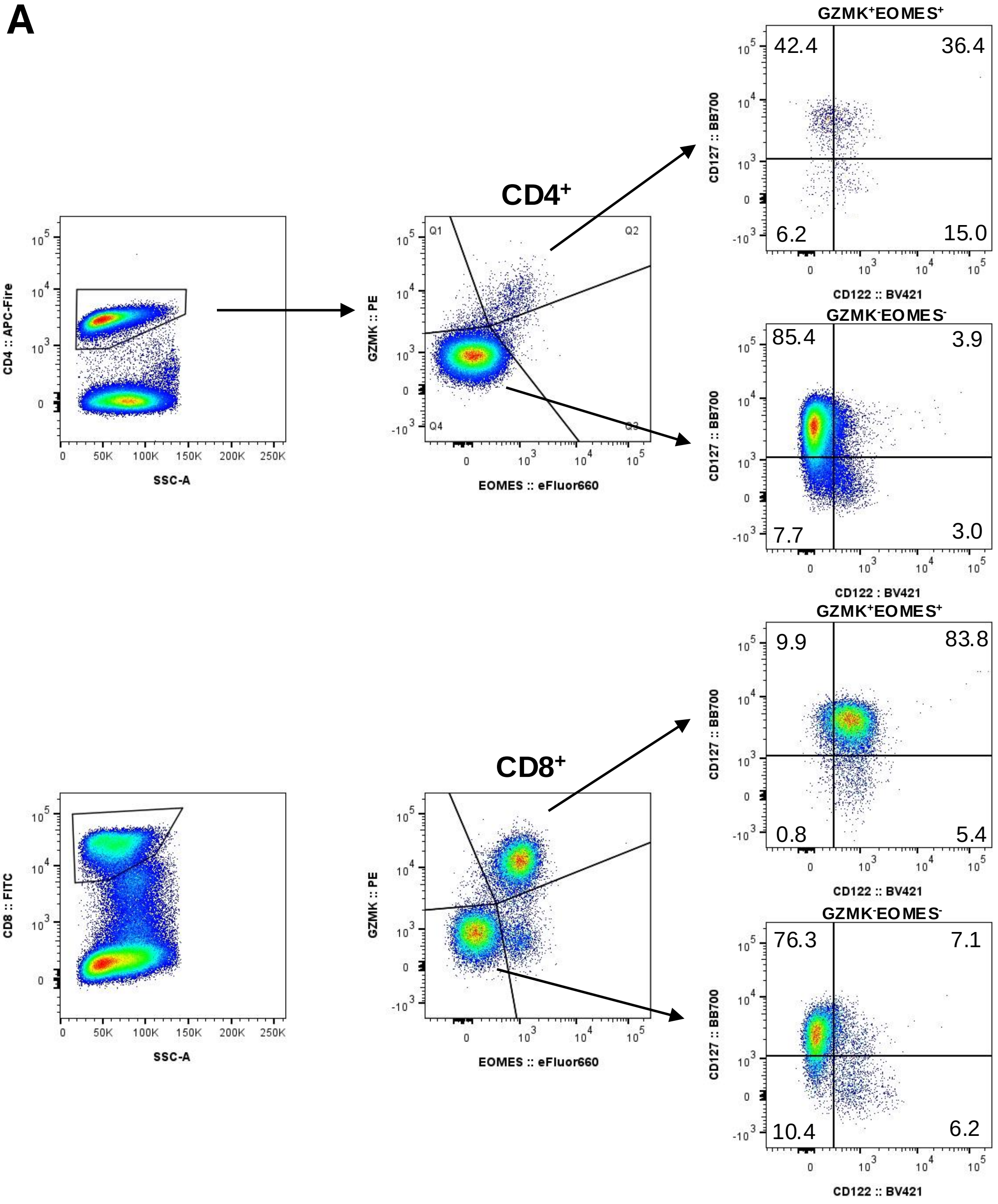

A. Primary FACS data showing ex vivo CD122 vs IL-7Rα expression on human blood CD4⁺ and CD8⁺T-cell subsets gated according to EOMES and GzmK expression.

sFigure 5: Gating strategy to analyse CD122 expression on T-cell subsets

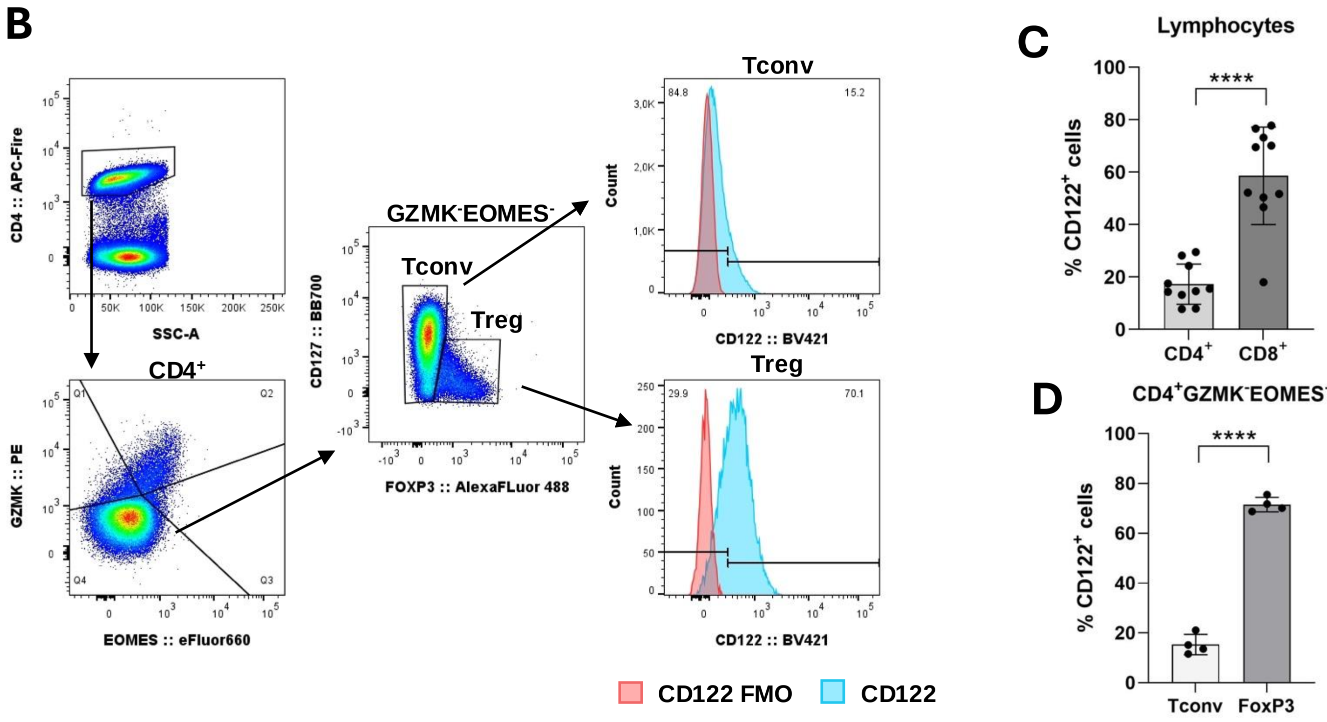

B. Gating strategy and primary FACS data showing ex vivo CD122 expression in Tconv and Treg. **C-D.** CD122 expression levels in the CD4⁺ and CD8⁺ cells gated on total lymphocytes (**C.**), and in Tconv and Treg gated on CD4⁺GzmK⁻EOMES⁻ (**D.**).

sFigure 6: Gating strategy to FACS-purify T-cell populations for survival assays

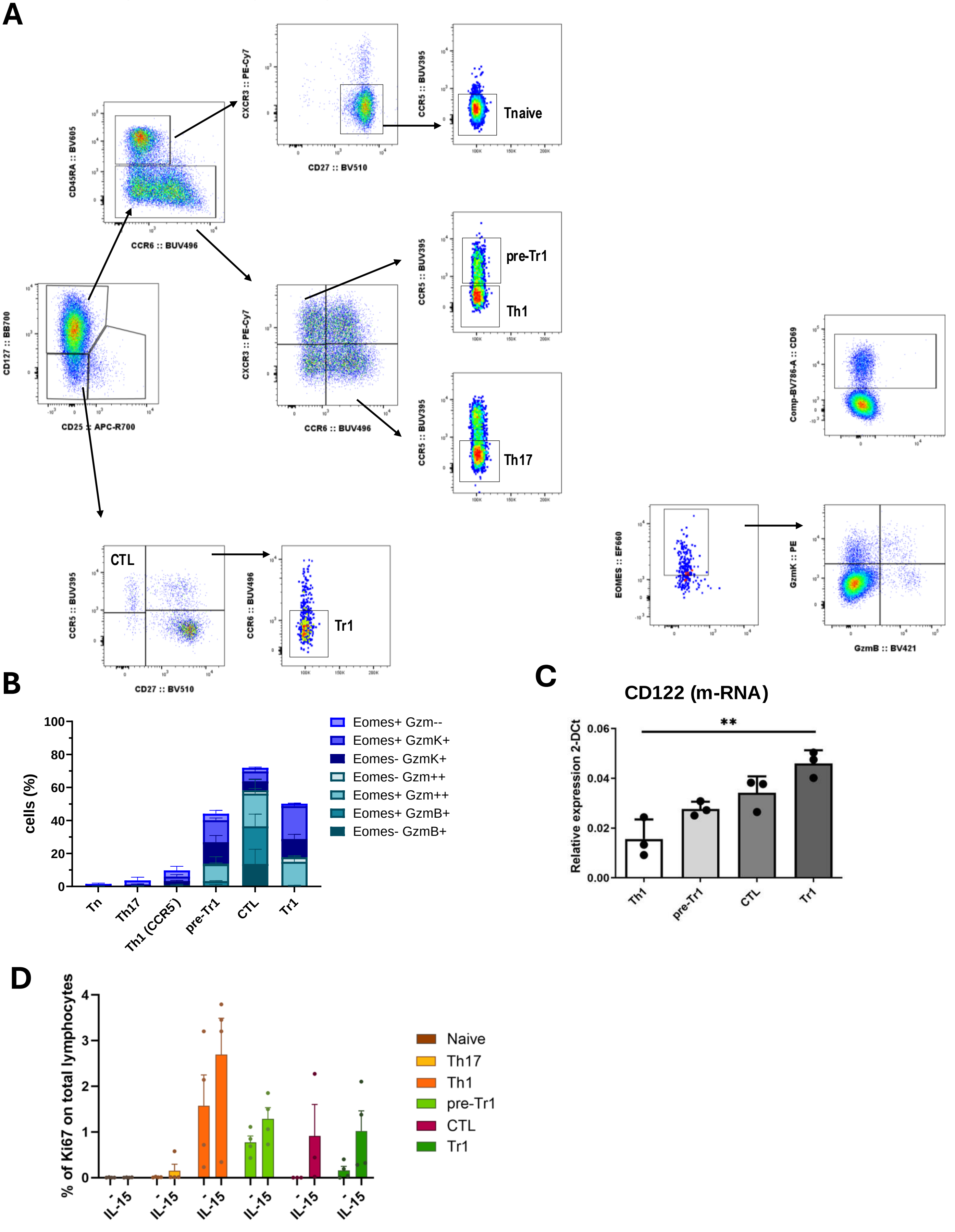

A Gating stategy to sort the indicated CD4^{+}cytotoxic and helper T-cell subsets **B**. Expression of EOMES, GzmK and GzmB in the CD4^{+}T-cell subsets gated as shown in panel **A** **C.** CD122 m-RNA expression in the indicated FACS-purified T-cell populations **D.** Ki67 expression after 4 days in FACS-purified CD4^{+}T-cell subsets according to the gating strategy (**A)** in the absence or presence of IL-15.

sFigure 7: Gating strategy to FACS-purify highly enriched Tr1-like cells for survival assays

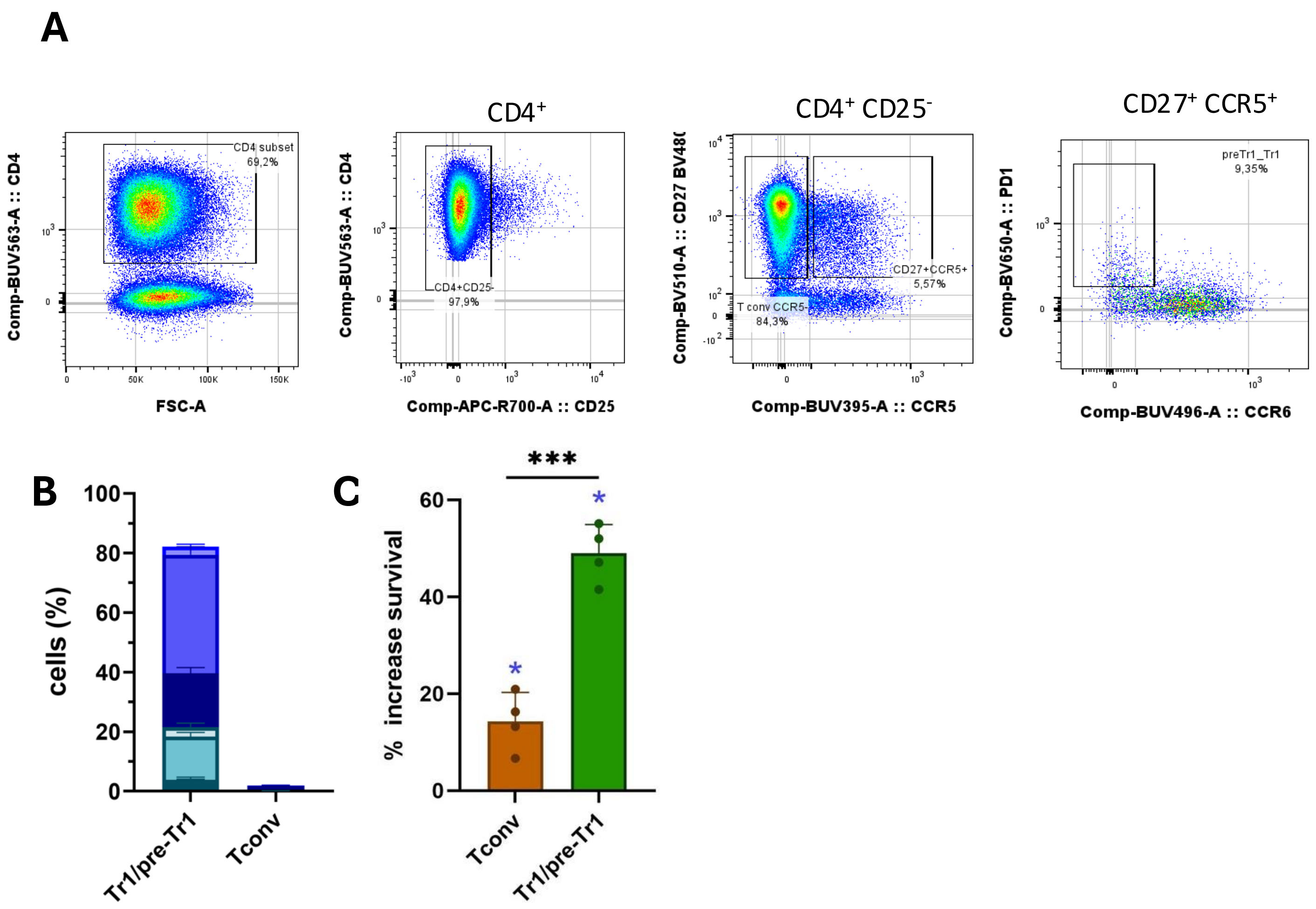

A. Gating strategy to obtain highly enriched Tr1- and pre-Tr1-like cells with anti-PD1 in healthy donor blood samples. **B.** EOMES, GzmK and GzmB expression patterns in gated PD1$^+$Tr1/pre-Tr1-enriched populations and CD4$^+$CD27$^+$CCR5$^-$ Tconv control cells. **C.** IL-15$^-$induced survival of FACS-purified Tr1/pre-Tr1-enriched populations and CD4$^+$CCR5$^-$ control cells was calculated by dividing the fractions of viable cells in the prensence and absence of IL-15 (n=4). (*) on top of the bars indicate statistically significant increased viability of cells stimulated with recombinant IL-15 as compared to medium alone. (***) on the line indicate statistically significant improved survival of preTr1/Tr1 cells as compared to Tconv with IL-15.

sTable 1: List of antibodies

Antibody	Fluorochrome	clone	Brand	cat. number
CD127	BB700	HIL-Tì7R-M21	Becton Dickinson	566398
CXCR3	PE-Cy7	1C6/CXCR3	Becton Dickinson	560831
CCR6	BUV496	11A9	Becton Dickinson	564659
CCR6	PE-Vio770	REA190	Miltenyi Biotec	130-117-685
CD4	BUV563	SK3	Becton Dickinson	566000
CD4	APC-Fire750	RPA-T4	Biolegend	300560
CD8	BUV805	SK1	Becton Dickinson	564912
CD8	FITC	HiT8a	Becton Dickinson	555634
CD8	VioGreen	BW135/80	Miltenyi Biotec	130-113-164
L/D	APC-H7		Becton Dickinson	565388
CD45RA	BV 605	HI100	Becton Dickinson	562886
CD69	BV786	FN50	Becton Dickinson	563834
CD3	BUV737	UCHT1	Becton Dickinson	612750
CCR7	PE-CF594	150503	Becton Dickinson	562381
CD161	PE-Cy5	DX12	Becton Dickinson	551138
CD25	APC-R700	2A3	Becton Dickinson	565106
CD27	BV480	L128	Becton Dickinson	566139
PD1	BV650	EH12.1	Becton Dickinson	564104
CCR5	BUV395	2D7/CCR5	Becton Dickinson	565224
FoxP3	AF488	259D	Biolegend	320212
GzmK	PE	SC56125	SantaCruz	GM6C3
EOMES	eFluor660	WD1928	eBioscience	50-4877-42
GzmB	BV421	GB11	Becton Dickinson	563389
Ki-67	BV711	B56	Becton Dickinson	563755
CD122	PE	Mik-β3	Becton Dickinson	554525
CD122	BV421	TU27	Biolegend	339010

sTable 2A Phenotype of gated T-cell subsets

	Phenotype (sFigure 1, 5B/D)
CD4	CD3$^+$CD4$^+$CD8$^-$
CD4 naive	CD3$^+$CD4$^+$CD8$^-$FOXP3$^-$EOMES$^-$RA$^+$CCR7$^+$
CD4 T_{CM}	CD3$^+$CD4$^+$CD8$^-$FOXP3$^-$EOMES$^-$RA-CCR7$^+$
CD4 T_{EM}	CD3$^+$CD4$^+$CD8$^-$FOXP3$^-$EOMES$^-$RA-CCR7-
Treg	CD3$^+$CD4$^+$CD8$^-$FOXP3$^+$
CD4 EOMES$^+$	CD3$^+$CD4$^+$CD8$^-$FOXP3$^-$EOMES$^+$
CD4 CTL	CD3$^+$CD4$^+$CD8$^-$FOXP3$^-$EOMES$^+$GzmK$^-$GzmB$^+$
Tr1	CD3$^+$CD4$^+$CD8$^-$FOXP3$^-$EOMES$^+$GzmK$^+$GzmB$^-$CCR6$^-$IL7R$^-$
pre-Tr1	CD3$^+$CD4$^+$CD8$^-$FOXP3$^-$EOMES$^+$GzmK$^+$GzmB$^-$CCR6$^-$IL7R$^+$
CCR6$^+$GzmK$^+$	CD3$^+$CD4$^+$CD8$^-$FOXP3$^-$EOMES$^+$ GzmK$^+$GzmB$^-$CCR6$^+$
CD8	CD3$^+$CD4$^-$CD8$^+$
CD8 naive	CD3$^+$CD4$^-$CD8$^+$RA$^+$CCR7$^+$
CD8 T_{CM}	CD3$^+$CD4$^-$CD8$^+$RA$^-$CCR7$^+$
CD8 T_{EM}	CD3$^+$CD4$^-$CD8$^+$RA$^-$CCR7$^-$
CD8 T_{EMRA}	CD3$^+$CD4$^-$CD8$^+$RA$^+$CCR7$^-$
CD8 GzmK$^+$	CD3$^+$CD4$^-$CD8$^+$EOMES$^+$GzmK$^+$GzmB$^-$
CD8 GzmB$^+$	CD3$^+$CD4$^-$CD8$^+$EOMES$^+$GzmK$^-$GzmB$^+$
CD8 DP	CD3$^+$CD4$^-$CD8$^+$EOMES$^+$GzmK$^+$GzmB$^+$
Tconv	CD4$^+$GZMK$^-$EOMES$^-$FOXP3$^-$
Treg	CD4$^+$GZMK$^-$EOMES$^-$CD127^{lo}FOXP3$^+$

sTable 2B Sorting strategies

sorted T-cell populations	Phenotype (sFigure 6A/7A)
CD4 naïve	CD4$^+$CD45RA$^+$CD27$^+$CXCR3$^-$CCR6$^-$CCR5$^-$
Th1	CD4$^+$CD45RA$^-$CD127$^+$CXCR3$^+$CCR6$^-$CCR5$^-$
Th17	CD4$^+$CD45RA$^-$ CD127$^+$CXCR3$^-$CCR6$^+$CCR5$^-$
pre-Tr1-enriched	CD4$^+$CD45RA$^-$CD127$^+$CXCR3$^+$CCR6$^-$CCR5$^+$
Tr1-enriched	CD4$^+$CD127-CD27$^+$CCR6$^-$CCR5$^+$
CTL-enriched	CD4$^+$CD127-CD27$^-$CCR5$^+$
highly pre-Tr1/Tr1-enriched	CD4$^+$CD25$^-$CD27$^+$CCR6$^-$CCR5$^+$PD1$^+$
Tconv	CD4$^+$CD25$^-$CD27$^+$CCR5$^-$
