# Supplementary material for: The Human Bone Marrow May Offer an IL‐15‐Dependent Survival Niche for EOMES+ Tr1‐Like Cells
Source: Eur J Immunol. 2025 May 16;55(5):e202451644. doi: 10.1002/eji.202451644 (PMC12082382; doi:10.1002/eji.202451644)
Supplement: Supplementary file 3 — Supporting information [file EJI-55-e202451644-s001.docx]

|  | Phenotype (sFigure 1, 5B/D) |
| --- | --- |
| CD4 | CD3^+^CD4^+^CD8^-^ |
|  |  |
| CD4 naive | CD3^+^CD4^+^CD8^-^FOXP3^-^EOMES^-^RA^+^CCR7^+^ |
| CD4 T_CM_ | CD3^+^CD4^+^CD8^-^FOXP3^-^EOMES^-^RA-CCR7^+^ |
| CD4 T_EM_ | CD3^+^CD4^+^CD8^-^FOXP3^-^EOMES^-^RA-CCR7- |
| Treg | CD3^+^CD4^+^CD8^-^FOXP3^+^ |
|  |  |
| CD4 EOMES^+^ | CD3^+^CD4^+^CD8^-^FOXP3^-^EOMES^+^ |
| CD4 CTL | CD3^+^CD4^+^CD8^-^FOXP3^-^EOMES^+^GzmK^-^GzmB^+^ |
| Tr1 | CD3^+^CD4^+^CD8^-^FOXP3^-^EOMES^+^GzmK^+^GzmB^-^CCR6^-^IL7R^-^ |
| pre-Tr1 | CD3^+^CD4^+^CD8^-^FOXP3^-^EOMES^+^GzmK^+^GzmB^-^CCR6^-^IL7R^+^ |
| CCR6^+^GzmK^+^ | CD3^+^CD4^+^CD8^-^FOXP3^-^EOMES^+^ GzmK^+^GzmB^-^CCR6^+^ |
|  |  |
| CD8 | CD3^+^CD4^-^CD8^+^ |
|  |  |
| CD8 naive | CD3^+^CD4^-^CD8^+^RA^+^CCR7^+^ |
| CD8 T_CM_ | CD3^+^CD4^-^CD8^+^RA^-^CCR7^+^ |
| CD8 T_EM_ | CD3^+^CD4^-^CD8^+^RA^-^CCR7^-^ |
| CD8 T_EMRA_ | CD3^+^CD4^-^CD8^+^RA^+^CCR7^-^ |
|  |  |
| CD8 GzmK^+^ | CD3^+^CD4^-^CD8^+^EOMES^+^GzmK^+^GzmB^-^ |
| CD8 GzmB^+^ | CD3^+^CD4^-^CD8^+^EOMES^+^GzmK^-^GzmB^+^ |
| CD8 DP | CD3^+^CD4^-^CD8^+^EOMES^+^GzmK^+^GzmB^+^ |
|  |  |
| Tconv | CD4^+^GZMK^-^EOMES^-^FOXP3^-^ |
| Treg | CD4^+^GZMK^-^EOMES^-^CD127^lo^FOXP3^+^ |

**sTable 2A Gating strategies**

**sTable 2B Sorting strategies**

| sorted T-cell populations | Phenotype (sFigure 6A/7A) |
| --- | --- |
|  |  |
| CD4 naïve | CD4^+^CD45RA^+^CD27^+^CXCR3^-^CCR6^-^CCR5^-^ |
| Th1 | CD4^+^CD45RA^-^CD127^+^CXCR3^+^CCR6^-^CCR5^-^ |
| Th17 | CD4^+^CD45RA^-^ CD127^+^CXCR3^-^CCR6^+^CCR5^-^ |
| pre-Tr1-enriched | CD4^+^CD45RA^-^CD127^+^CXCR3^+^CCR6^-^CCR5^+^ |
| Tr1-enriched | CD4^+^CD127-CD27^+^CCR6^-^CCR5^+^ |
| CTL-enriched | CD4^+^CD127-CD27^-^CCR5^+^ |
|  |  |
| highly pre-Tr1/Tr1-enriched | CD4^+^CD25^-^CD27^+^CCR6^-^CCR5^+^PD1^+^ |
| Tconv | CD4^+^CD25^-^CD27^+^CCR5^-^ |
